# News and Notes

**Published:** 1909-03

**Authors:** 


### NEWS AND NOTES

The Seventh District Medical Society held its third annual session at Marietta, Ga., March 10, 1909. The following are the officers: President, R. P. Cox, M. D., Rome, Ga.; vice-president, C. F. McLain, Calhoun, Ga.; Secretary and treasurer, H. L. Erwin, Dalton, Ga.

---

At a recent meeting of the Board of Trustees of Grady Hospital, the following physicians were elected: Dr. G. P. Huguley, assistant to Dr. J. C. Olmsted; Dr. F. G. Hodgson, assistant to Dr. Willis Jones; Dr. Archibald Smith, assistant to Dr. Geo. H. Noble; and Dr. W. E. Yankey, assistant to Dr. L. C. Fischer, Dr. Westmoreland was elected surgeon to succeed Dr. W. B. Armstrong, deceased.

---

The medical class of 1879 of the Atlanta Medical College, gathered at the Piedmont hotel March 5th at a banquet tendered them by Dr. H. B. Stewart, of Fountain Inn, S. C. Dr. W. S. Elkin, Dr. Willis Westmoreland and Dr. O. W. Calhoun were invited. The others present at the dinner were: Dr. J. L. Selman, Douglasville; Dr. W. J. Adair, of Rockmart; Dr. R. H. Taylor, of Griffin; Dr. B. F. Braselton, of Weatherford, Tex.; Dr. W. J. Chapman, of Inman, S. C.; Dr. J. M. Spinks, of Rockmart; Dr. H. D. Allen, of Milledgeville, and Dr. H. B. Stewart, of Fountain Inn, S. C.

---

The department of agriculture has received complaint as to the existence of glanders among the horses and mules in certain portions of Monroe county, and fears are expressed that the disease will spread, causing the death of much valuable stock.

---

Mrs. William K. Vanderbilt, Sr., has given more than $1,000,000 for the erection of four modern tenements for persons suffering with tuberculosis. The buildings are to be operated in connection with Dr. Henry L. Shively's tuberculosis clinic, of the Presbyterian hospital, and are to be known as the Shively Sanitary Tenements. A moderate rental will be charged tenants.

---

### SAVING DOCTORS.

Knicker—"There are plenty of books telling how to save life while waiting for the doctor."

Bocker—"Yes. What we need is one telling the young doctor how to save life while waiting for the patient."—*Harper's Bazar.*

---

### REGULAR MEETING FULTON COUNTY MEDICAL SOCIETY, CARNEGIE LIBRARY, FEBRUARY 18, AT 8 P. M.

---

REPORTED BY R. R. DALY, M. D.

---

Dr. Strickler, the president, presiding.

Dr. Roy read his paper upon "Lachrymal Stenosis in Children."

Dr. Stirling said he had seen several cases of infantile stenosis and epiphira, all of which recovered under massage, collyria and expectant treatment. He never had to operate in any way. He told of an absence of one canaliculus in an adult. The slitting and probing are to be avoided as a rule and used only after every other means have been exhausted. Sometimes he passes a hollow needle into the duct and injects through it what he requires. This leaves no scar or deformity. In old chronic cases of epiphira, he advocates the removal of the tear sac.

Dr. Daly said his experience had been along the line of massage rather than operation in children. The expectant treatment was aided frequently by completion of development of the parts.

Dr. Roy, in closing, said he hoped to interest the obstetrician in the subject so that the natural worry of the mother might be relieved by the assurance that the child would get well.

He believes that most of the cases are due to lack of development.

Dr. Kime gave an interesting paper upon "Some Experiences in Surgery of the Appendix and Report of Cases." This paper appears elsewhere in *The Journal-Record.*

Dr. Willis Jones said in discussion that only about a fourth of his cases showed the appendix in normal position and with normal peritoneal covering. He asked Dr. Kime's experience in this regard.

In a recent tubercular case, he found great pain and considerable pus with disintegration of the appendix. Case died. There was no temperature, but high pulse.

Another case had violent pain at appendix, high pulse and no temperature. He hasn't had as good fortune as Dr. Kime.

Dr. Davis congratulated Dr. Kime upon his success with serious and complicated cases and regrets that his own experience has not been so gratifying. He commented upon the injudicious use of purgatives because of the increased peristaltic wave they set up may cause rupture in lower part of intestine.

Dr. Hardin complimented Dr. Kime upon his paper and his successful surgery in getting his cases operated upon early. He thinks that turning patients flat on abdomen in transit to the hospital is better than Fowler's position, as well as after operation.

Dr. Kime in closing said that he believes Fowler's the better position because the secretions are kept away from the diaphragm which part of the peritoneum absorbs poisons more readily.

Dr. Visanska reported a case of lobar pneumonia in 6 year old child with hyperaesthesia of the diaphragm and stiffness of neck and hips. The diagnosis was obscure at first.

Another child had suspected trouble in the appendix, but developed posterior pneumonia on right side.

Another case, 17 months old, presented diphtheria with no special constitutional symptoms. It seemed a little sick and he looked in the throat only as a matter of routine. The membrane was entirely upon posterior pharyngeal wall, and not on tonsils or pillars at all. He suggests that the toxines were not absorbed because of the smaller lymphatic supply to the part involved, this accounting for lack of constitutional symptoms.

### MEETING FULTON COUNTY MEDICAL SOCIETY HELD AT CARNEGIE LIBRARY, MARCH 3, 1909.

Dr. Strickler, president, in the chair.

Dr. Amster read an interesting paper upon "The Diagnostic Value of Test Meals in Gastro-Intestinal Diseases."

Dr. Andrews said in discussing it, that many of the tests are not so easy as the essayist stated. The Schmidt and Strasburger diet and test take considerable time, often lasting several days. The value of test meals depend upon whether they have been thoroughly cooked. Muscular fibre is very differently manifested in the stools when its surrounding tissue has been so broken up by cooking as to allow the digestive processes to go on from the same fibre when it is ingested partly cooked or raw.

Fermentative tests have to be made for undigested starch and biologic examinations are necessary to determine the intestinal flora. Too much importance cannot be placed upon this subject and if the tests are too quickly or only partially made, valuable information is lost and the conclusions may be misleading.

As to occult blood great care is necessary to prevent small haemorrhoids giving it in the stools or irritation of the stomach tube causing it to appear in the gastric contents from the injured gums or throat.

Dr. Niles said that many patients had such a horror of the stomach tube that whenever he could, he gave a retention meal and then palpation was made in 3 1-2 hours. If a gurgle or splash resulted, it meant myasthenia.

He called special attention to attempting through subjective methods to diagnose hyperacidity. The burning sensations do not always mean too much acid by any means.

Accurate methods should be employed to prevent grave error. Too much bi-carbonate of soda is given anyway.

Dr. Amster said in closing that his paper was intended to be of clinical value and not an attempt to go exhaustively into scientific tests. When he used the quick methods and advised them, he meant to find by them the right track upon which to pursue such further examinations as were indicated. He wished to stimulate everyone to make such examinations before completing a diagnosis and beginning a treatment.

Dr. McRae spoke on the subject of "Prostatectomy."

Dr. Ballenger said that there was reasonable ground for the advocacy and choice of both the supra-pubic and the perineal methods; the large adenomatous glands are better removed above while small ones come more readily from below. As a preliminary operation, he frequently procures supra-pubic drainage by thrusting in a large trocar and canula, (as advocated by Belfield) introducing a soft catheter through the canula and then removing the canula, leaving the catheter in place. This drains the bladder until the conditions improve and the patient is better able to stand the operation.

Dr. Willis Jones agrees with Dr. McRae that the operation should always be done to save constant catheter life and be done as early as this condition seemed imminent. The supra-pubic method seems to have from two to three times the mortality of the sub-pubic, but its results are better when recovery occurs. There is no pain on sitting and the urethra is in far better condition.

Dr. Hancock suggested the dividing the operation into two sittings. He suggests the anaesthetic may be the cause of death.

Dr. Goldsmith described Dr. Young's methods and says it is marvelous to see him extract the gland through the perinaeum even with the aid of his special tractors and retractors. Young has a high table so that the operative field is directly before him where he doesn't need to bend and tire himself to see what he is doing. Dr. Goldsmith exhibited a prostate he had removed the previous day. The first part of the operation was sub-pubic and only a small part of the gland could be secured. He thought he had enough to give relief. A few hours later, the patient was in greatest distress, totally unable to void urine. He hastily went down supra-pubically and removed a mass five times as large as the entire gland. Relief was complete and the patient is doing well.

Dr. McRae in closing says that all the worst cases are done by the supra-pubic method which doubtless accounts for much of its apparently greater mortality.

The anaesthetic should be gas-oxygen and even then there is trouble in getting complete relaxation.

### MEETING FULTON COUNTY MEDICAL SOCIETY HELD AT CARNEGIE LIBRARY, MARCH 18, AT 8 O'CLOCK

Dr. Strickler, the president, presiding.

Dr. Phinizy Calhoun presented a case of cystic degeneration of the lachrymal sac that had caused exopththalmus and partial loss of vision due probably to neuritis. Several years before, large polypi had been removed from each naris. They recurred and were removed again a few weeks ago.

The operation for the cyst consisted in opening at the eyebrow and shelling out the tumor which fortunately came easily. The recovery was rapid.

Dr. Kime exhibited a large multilocular cyst removed from woman 79 years old. Passed change of life at 48. Had 12 children, youngest 32, no miscarriage. First enlargement of cyst came 8 months ago. Previous to operation, palpation at different parts of the abdomen had shown different degrees of density. The fluid, nearly three gallons was drawn from the cyst under different degrees of pressure. The Mayos advised removal of the cyst entirely because about 4 per cent. of those drained first, are followed by cancer. Dr. Kime preferred draining in this instance because of the danger of collapse if the large growth were suddenly lifted from the abdominal vessels.

Dr. Baird read his paper on "Acute Lobar Pneumonia," which appears elsewhere in the *Journal.*

Dr. Duncan said he agreed almost completely with the essayist, but would proceed with caution in the use of phenacetine and all the coal tar derivatives. He wants to use veratrum in young and robust subjects and usually combines it with some opiate. He always uses quinine, dover's powder or capsecum. He agrees that glycerine and clay that "comes in cans" is of no use. Sometimes sinapisms and jackets are good especially where there is pleuritis. He doubts the value of the general use of whiskey.

Dr. Roberts said that the line of treatment should be considered from three standpoints:

1.—That of cough and infection.

2.—That of symptoms of essential fever.

3.—That of the cause.

We don't know enough of why the germs cause the condition to enable us to attack the activity successfully in that way. We must use the knowledge we have if the essential fever as it shows its activities in our experience and treat along that line. It is manifestly a disease of obstructed circulation with all that applies and we must give our aid and stimulation to relieving the obstruction and increasing the force of the circulatory apparatus.

Dr. Baird in closing laid stress again upon the value of phenacetine and said that its intelligent use by the careful clinician was attended with no danger whatsoever. Two grains every 2 to 4 hours was his dosing and he watched this. The heat itself we know to degenerate muscular and nervous tissues and that the heart suffers in both these ways from high temperature. High temperature does not destroy the germs except by injuring the patient and we as physicians have no right to stand by and let the battle rage in our patient's body. Phenacetine will reduce fever. will give the patient comfort and rest and will assist him in recovering. Whether it has any affects upon the toxines he doesn't know. He confidently expects that in the next decade, there will be such advances in biology as to give us knowledge of the germ life, the propagation if its toxines and a suitable means for combatting their evil effects.

Dr. P. Calhoun read an exhaustive and interesting paper upon the "Present Status of the Ophtholmo-Tuberculin Reaction of Calmette."

Statistics for and against this method of diagnosis were presented together with the recently expressed opinions of many eminent opththolmologists sent to the writer. His conclusions were that it might have some value but should never be given except in an eye one was sure of being normal. He would use it himself in such cases. It has no opththalmological value.

Dr. Lokey said he has been treating several cases lately in connection with the family doctor and is not satisfied with the reaction. It is not decisive. The statistics in America are not reliable in his judgment because we don't hear of the unfavorable cases. Among the negroes and the Russian Jews we get an over-positive reaction because, doubtless, of the ill nourished condition of many of these people.

Dr. Paullin spoke at length upon the method of securing the tuberculin. It is a question in any injection as to whether we are using a potent toxine. There is no standard of judging toxicity. Koch's old tuberculin may vary 100 per cent. in the rabbet test and this takes several days to accomplish. He is not opposed to the skilled use of tuberculin.

Dr. Wheeler is opposed to the use of uncertain and sometimes dangerous method of examination. He sees nothing to be gained by it.

Dr. Daly said that the clinical methods of examination were so much better than they used to be that one could rely confidently upon them and the laboratory assistance without endangering an eye with what often proved harmful. He had recently seen two eyes badly inflamed by the Calmette injection. He would not introduce it into his own eye if he suspected tuberculosis, but would prefer to wait and keep his eyes in good order even if he had the disease. If he did not have it the eyes were never endangered.

Dr. Gaines said that the reaction is so doubtful that we are no better off in diagnosis than we were before Calmette gave out his theory. The general practitioner doesn't pretend to be sure an eye is normal and then many of his cases do not have normal eyes and other methods must be used. The best clinicians are coming back to the hypodermatic plan so far as tuberculin is concerned. There the doses can be graduated carefully, the temperature noted and the presence or absence of moist rales at the primary foci be determined. In this clinical way, diagnosis may be assured.

Dr. Ballenger said he had introduced the tuberculin into the urethra instead of the eye as here it is less likely to do harm and is yet in touch with mucous membrane. The reaction when gained is slight irritation as if the urine were concentrated and a slight redness at the meatus.

Dr. Calhoun said he was in no sense an advocate for the method, but wanted to present the matter to the society in all its phases.

Dr. Kime told of administering to a man 70 years old 1-100 grain of scopolamine and 1-8 grain of morphine at 3 o'clock. At 6 o'clock there was active delirium, the man insisting upon getting out of bed. He gave 5 drops of veratrum hypodermacally and catheterized the patient. In an hour he was quiet. Dr. Kime asked if others had had any similar experience with the small amount of scopolamine.

Dr. Sellman said that he used the drug frequently as a preliminary to anaesthesia and sometimes, there was muttering delirium but nothing active.

Dr. Willis Jones reported a case of stone in the kidney. The patient was a woman 21, married and with two children.

All her life she had had recurring attacks of pain in the right side of the abdomen, frequently accompanied with hematurea, sometimes lasting several days.

Ten days ago her family doctor said he could feel and hear stone in the kidney. Dr. Jones was called in and doubted the doctor's statement till he himself heard the crepitance. The haemorrhage after manipulation was profuse. Strangely enough the lady's chief pleasures which were horseback riding and skating never caused her either distress or haematurea.

Four days ago, he operated and removed some six or eight large calculie which he exhibited. He had heard them sliding upon each other in the kidney under manipulation. They were of different formation, some were phosphatic and others uric acid deposits.

Dr. J. L. Campbell related a case of prostatic colic if one will admit the term. A man had several attacks of pain recurring in right side of prostate. On palpation a good sized lump appeared where it might be an enlarged seminal vesical. The manipulation amounted to massage. Patient was told to bring sample of urine next morning. He appeared at that time and presented a small stone about the size of a grain of wheat which he said he had passed through the urethra. The prostate trouble at once disappeared and patient made a perfect recovery. This calculus probably stopped one of the seminal ducts and was dislodged by massage.

